# Binding kinetics, bias, receptor internalization and effects on insulin secretion in vitro and in vivo of a novel GLP‐1R/GIPR dual agonist, HISHS‐2001

**DOI:** 10.1111/dom.16652

**Published:** 2025-08-20

**Authors:** Yusman Manchanda, Ben Jones, Gaelle Carrat, Zenouska Ramchunder, Piero Marchetti, Isabelle Leclerc, Rajamannar Thennati, Vinod Burade, Muthukumaran Natarajan, Pradeep Shahi, Alejandra Tomas, Guy A. Rutter

**Affiliations:** ^1^ Section of Cell Biology and Functional Genomics, Department of Metabolism, Digestion and Reproduction, Faculty of Medicine Imperial College London London UK; ^2^ Section of Endocrinology and Investigative Medicine, Department of Metabolism, Digestion and Reproduction, Faculty of Medicine Imperial College London London UK; ^3^ Department of Experimental and Clinical Medicine Pancreatic Islets Laboratory, University of Pisa Pisa Italy; ^4^ Centre de Recherche du Centre Hôspitalier de l'Université de Montréal (CRCHUM) et Faculté de Médecine, Université de Montréal Montréal Quebec Canada; ^5^ Sun Pharmaceutical Industries Limited Vadodara Gujarat India; ^6^ Lee Kong Chian School of Medicine, Nanyang Technological University Singapore; ^7^ Research Institute of the McGill University Health Sciences Centre Montreal Quebec Canada

**Keywords:** antidiabetic drug, antiobesity drug, beta cell function, GLP‐1 analogue, insulin secretion

## Abstract

**Aims:**

The use of incretin analogues has emerged as an effective approach to achieve both enhanced insulin secretion and weight loss in Type 2 diabetes (T2D) patients. Agonists which bind and stimulate multiple receptors have shown particular promise. However, off‐target effects remain a complication of using these agents, and modified versions with optimised pharmacological profiles and/or biased signalling are sought.

**Materials and Methods:**

Ligand synthesis was achieved using standard solid‐phase techniques. Assessments of GLP‐1R‐binding kinetics, G protein recruitment and receptor internalisation were performed using biochemical and imaging approaches. Insulin secretion was measured in purified mouse and human islets, and drug efficacy was assessed in hyperglycaemic *db/db* mice.

**Results:**

We describe the synthesis and properties of a molecule which binds to both glucagon‐like peptide‐1 (GLP‐1) and glucose‐dependent insulinotropic polypeptide (GIP) receptors (GLP‐1R and GIPR) to enhance insulin secretion. HISHS‐2001 shows increased affinity at the GLP‐1R, as well as a tendency towards reduced internalisation and recycling at this receptor versus FDA‐approved dual GLP‐1R/GIPR agonist tirzepatide. HISHS‐2001 also displayed significantly greater bias towards cAMP generation versus β‐arrestin 2 recruitment compared to tirzepatide. In contrast, G_α_s recruitment was lower versus tirzepatide at the GLP‐1R, but unchanged at the GIPR. Administered to obese hyperglycaemic *db/db* mice, HISHS‐2001 increased circulating insulin whilst lowering body weight and HbA1c with similar efficacy to tirzepatide at substantially lower doses.

**Conclusion:**

HISHS‐2001 represents a novel dual receptor agonist with a promising pharmacological profile and actions. Future clinical studies will be needed to assess the safety and efficacy of this molecule in humans.

## 1 INTRODUCTION

Almost one third of North Americans are obese and two thirds are overweight.^1^ Type 2 diabetes (T2D), driven in part by these increases in obesity in recent years, now affects almost 1 in 10 of the population of westernized societies,^2^ while the total number of cases worldwide is expected to rise to >600 million by 2045.^3^

Originally identified as effective treatments for T2D,^4^, ^5^ GLP‐1R agonists have proven effective in recent years in reducing both body weight and hyperglycaemia.^6^ Dual and triple receptor agonists are proving even more effective,^7^, ^8^ with weight loss of 20% or more now achievable with the FDA‐approved GLP‐1R–GIPR co‐agonist, tirzepatide.^9^ These agents bind to receptors in the endocrine pancreas, notably on the pancreatic beta cell, potentiating the effects of glucose to stimulate insulin secretion, as well as to receptors in the brain, heart, kidney, adipose tissue and immune cells^10^, ^11^, ^12^ to promote energy expenditure and reduce appetite, amongst other beneficial effects. Dual incretin receptor agonists appear to deliver superior effects both through enhanced activation of insulin secretion^13^ but also centrally as a result of improved anti‐emetic effects versus agonists which act through the GLP‐1R alone.^14^

## 2 MATERIALS AND METHODS

### 2.1 Animal maintenance and ethical approvals

Studies at Imperial College London were approved by the Animal Welfare and Ethical Review Body according to the UK Home Office Animals Scientific Procedures Act, 1986 (Project Licence PA03F7F0F to Isabelle Leclerc). All in vivo procedures at Sun Pharmaceuticals Inc. were approved by the Institutional Animal Ethics Committee (IAEC #608 and 650). Use of human islets was approved by the National Research Ethics Committee London (Fulham), Research Ethics Committee No. 07/H0711/114, and by relevant national and local ethics committees including, where required, consent from next of kin. Human islet donor details are provided in Table S1.

### 2.2 Synthesis of HISHS‐2001

Briefly, the parent peptide was synthesized by conventional solid‐phase methods with Rink Amide as the starting resin used for synthesis. Following Fmoc de‐protection of Rink Amide resin using piperidine in dimethylformamide, Fmoc‐Ser(tBu)OH coupling with Rink Amide was performed using N,N'‐Diisopropylcarbodiimide (DIPC) and 1‐Hydroxybenzotriazole (HOBt) as coupling agents to yield Fmoc‐Ser(tBu)‐Resin. Uncoupled amino groups were capped after every amino acid coupling by acetylation. Next, selective de‐blocking of the amino group of Fmoc‐Ser(tBu)‐Rink Amide Resin was performed using piperidine, followed by coupling with Fmoc‐Pro‐OH using HOBt and DIPC to yield Fmoc‐Pro‐Ser(tBu)‐Rink Amide Resin. The above steps, that is, selective capping, deblocking of Fmoc protection of the amino acid attached to the resin and coupling of the next amino acid residue in sequence with Fmoc/Boc‐protected amino groups, were repeated for the remaining 37 amino acid residues. Once all amino acids are substituted on Rink Amide Resin, the 20th amino acid, that is, Lys, was selectively deprotected and attached to the side chain. Finally, the crude compound was deprotected and cleaved from the resin using trifluoroacetic acid (TFA)/ethanedithiol/triisopropyl silane (TIPS) mixture and purified through Preparative high performance liquid chromatography using Phenyl Silica column and mobile phase (ammonium format buffer, aq. acetic acid, phosphate buffer). Fractions with purity above 98% (single major impurity limit NMT 0.5%) were pooled and desalted, and the desalted solution was filtered (0.2 μm filter), and solvent was distilled on rotavapor at 35–40°C under vacuum and lyophilised for ~120 h.

### 2.3 Cell culture

HEK293 cells stably expressing human GLP‐1R or human GIPR, each self‐labelling protein tag (SNAP)‐tagged at their N‐termini (Cisbio), were cultured in Dulbecco's Modified Eagle's Medium (DMEM) medium (Gibco), 10% fetal bovine serum (FBS) and 1% penicillin/streptomycin. DiscoverX GLP‐1R‐β‐arrestin 2 cells were cultured in the manufacturer's recommended medium.

Rat insulinoma INS‐1832/3 cell lines were cultured in Roswell Park Memorial Institute (RPMI) 1640 medium (Gibco) supplemented with 10% FBS, 1% penicillin/streptomycin, 1 M 4‐ (2‐hydroxyethyl)‐1‐piperazineethanesulfonic acid (HEPES) buffer, 100 mM sodium pyruvate, and 0.05 mM β‐mercaptoethanol. The cell lines used were INS‐1832/3 GLP‐1R^−/−^ and INS‐1832/3 GIPR^−/−^ (kind gifts from Dr. Jackeline Naylor, MedImmune/AstraZeneca^15^), and INS‐1832/3 stably expressing SNAP‐tagged human GLP‐1R (generated *in house* and previously described^16^).

Cell lines were kept in an incubator at a temperature of 37°C and at a CO_2_ concentration of 5%.

### 2.4 cAMP assays

HEK293‐SNAP‐GLP‐1R, HEK293‐SNAP‐GIPR or GLP‐1R‐β‐arrestin 2 cells were seeded overnight in 12‐well plates. On the day of the assay, cells were resuspended and dispensed into white, 96‐well plates containing the indicated concentration of agonist prepared in serum‐free medium containing 0.1% bovine serum albumin (BSA). After a 30‐min stimulation, cAMP detection reagents (cAMP dynamic kit, Cisbio) were added, and the plate was read 60 min later using an HTRF‐compatible plate reader. Data were analysed using three‐parameter logistic fitting (GraphPad Prism 9.0) and normalised to the maximal fitted response of the reference ligand (GLP‐1 or semaglutide) for presentation purposes.

### 2.5 β‐Arrestin 2 recruitment assays

GLP‐1R‐β‐arrestin 2 cells were seeded overnight in a 12‐well plate. On the day of the assay, cells were resuspended and dispensed into white, 96‐well plates containing the indicated concentration of agonist prepared in serum‐free medium containing 0.1% BSA. After a 30‐min stimulation, β‐arrestin detection reagents (DiscoverX) were added and the plate was read 60 min later by luminescence. Data were analysed using three‐parameter logistic fitting (GraphPad Prism 9.0) and normalised to the maximal fitted semaglutide concentration for presentation purposes.

### 2.6 Calculation of bias

Bias between cAMP and β‐arrestin 2 was determined using a modified operational model of agonism. Concentration response data were fitted with previously described equations^17^ to derive transduction ratios (*τ*/KA) for each agonist and pathway. Log (*τ*/KA) values were normalised by subtracting log (*τ*/KA) for semaglutide in each pathway, giving Δlog (*τ*/KA), and to determine the bias between the two pathways, Δlog (*τ*/KA) values were subtracted, yielding ΔΔlog (*τ*/KA). This value is referred to as ‘bias factor’ in the text.

### 2.7 GLP‐1R binding kinetics

HEK293‐SNAP‐GLP‐1R cells were seeded overnight in 12‐well plates. On the day of the assay, cells were labelled with 40 nM Lumi4‐Tb (Cisbio) in complete medium for 30 min. After washing, labelled cells were preincubated for 20 min in Hanks' Balanced Salt Solution (HBSS) supplemented with 0.1% BSA and a metabolic inhibitor cocktail (10 mM NaN_3_, 20 mM 2‐deoxyglucose) to inhibit GLP‐1R endocytosis that would otherwise influence binding assays. Ligand binding was then detected in real time using time‐resolved Förster resonance energy transfer (TR‐FRET) using a Flexstation 3 plate reader (Molecular Devices). Ligand conditions included four or more concentrations of unlabelled HISHS‐2021 or tirzepatide in combination with 10 nM exendin(9–39)‐FITC, and exendin(9–39)‐FITC alone at four concentrations to determine its own kinetic parameters. Plate reader settings included 340 nm λexcitation, 520 nm (cut‐off 495 nm) and 620 nm (cut‐off 570 nm) λemission, 400 μs delay and 1500 μs integration time. Association and dissociation rate constants were calculated using the kinetics of competitive binding algorithm in GraphPad Prism 9.0.

### 2.8 G_α_s recruitment by NanoBiT complementation assay

INS‐1832/3 GLP‐1R^−/−^ and GIPR^−/−^ cells were seeded onto 6‐cm dishes the day before transfection and transfected the following day using Lipofectamine 2000 (Thermo Fisher Scientific) with 1 μg plasmid DNA to 2 μL Lipofectamine 2000 ratios. Cells were transfected with 1.7 μg GLP‐1R‐SmBiT or 1.7 μg GIPR‐SmBiT (cloned *in house*) and 1.7 μg G_α_s‐LgBiT (gift from Prof Nevin Lambert, Medical College of Georgia, USA). Four hours prior to the assay, the cell media was replaced with RPMI supplemented with 3 mM glucose. Cells were then washed with phosphate buffered saline (PBS), detached with ethylenediaminetetraacetic acid (EDTA), and resuspended in HBSS buffer, after which Nano‐Glo® LCS Dilution Buffer and Nano‐Glo® LCS Live Cell Substrate were added, according to the manufacturer's protocol (Promega), and a baseline reading was taken for 8 min. Cells were subsequently stimulated with the indicated concentration of agonist for 30 min, with a reading taken every 30 s. The luminescence (RLU) measured for each agonist was normalised to the baseline reading and to the vehicle luminescence reading. The AUCs were calculated and a dose–response curve generated in GraphPad Prism 9.0.

### 2.9 Lipid nanodomain isolation by membrane fractionation

GLP‐1R recruitment into lipid nanodomains was measured using INS‐1832/3 SNAP‐GLP‐1R cells as follows: 2 × 10^6^ cells were seeded on 6‐cm dishes the day before fractionation. On the day of fractionation, cells were stimulated with 100 nM agonist for 2 min, washed with ice‐cold PBS and osmotically lysed on ice in lysis buffer (20 mM Tris–HCl, pH 7, 1% protease inhibitor [Roche] and 1% phosphatase inhibitor [Sigma‐Aldrich]). Lysates were passed through 21‐gauge needles and ultracentrifuged at 41 000 rpm at 4°C for 1 h. Pellets were resuspended in PBS supplemented with 1% Triton‐X100, 1% protease inhibitor and 1% phosphatase inhibitor, incubated under rotation at 4°C for 30 min and ultracentrifuged again at 41 000 rpm at 4°C for 1 h. The detergent‐soluble membrane fractions (DSM, supernatants) were removed, and the detergent‐resistant membrane fractions (DRM, pellets) resuspended in 1% sodium dodecyl sulphate (SDS) with 1% protease inhibitor and 1% phosphatase inhibitors, sonicated, centrifuged at 1300 rpm at 4°C for 5 min and stored at −20°C for Western blot analysis. The DRMs and DSMs were diluted 1:1 with 2× Tris ‐Borate ‐ EDTA (ethalinediamine tetracetic acid) urea buffer (200 mM Tris–HCl, pH 6.8, 5% w/v SDS, 8 M urea, 100 mM dithiothreitol and 0.02% w/v bromophenol blue), incubated at 37°C for 10 min and resolved by SDS–polyacrylamide gel electrophoresis (10% acrylamide gels). Protein transfer to polyvinylidene fluoride membranes (Immobilon‐P, 0.45‐μm pore size, IPVH00010, Merck) was achieved using a wet transfer system (Bio‐Rad). Blotted membranes were blocked in 5% skimmed milk in TBS‐Tween buffer, incubated with primary rabbit anti‐SNAP monoclonal antibody (1:1000, New England Biolabs), followed by secondary horseradish peroxidase (HRP)‐conjugated anti‐rabbit antibody (1:2000, Invitrogen) in the same buffer and developed using the Clarity Western enhanced chemiluminescence substrate system (1705060, Bio‐Rad) in a Xograph Compact X5 processor. Membranes were then stripped in stripping buffer (20 g SDS, 7.6 g Trizma‐Base, pH 6, supplemented with 1% β‐mercaptoethanol) at 50°C for 15 min, blocked as above, and incubated with rat primary anti‐flotillin antibody (2.5 μg/μL, BioLegend), followed by secondary HRP‐conjugated anti‐rat antibody (1:2500, Sigma‐Aldrich). Specific band densities were quantified using Fiji ImageJ v1.53c. SNAP bands were normalized to flotillin and expressed as fold‐change over vehicle.

### 2.10 GLP‐1R internalisation and recycling assays

*GLP‐1R internalization*: INS‐1832/3 SNAP‐GLP‐1R cells were seeded in 14‐mm microwell glass bottom MatTek dishes (MatTek Life Sciences). Prior to imaging, the cells were labelled with SNAP‐Surface® 549 (New England Biolabs) for 20 min in full media. Cells were then washed and imaged in Live Cell Imaging Solution (Thermo Fisher Scientific) by time‐lapse spinning disk microscopy using a Nikon Eclipse Ti spinning disk microscope with an ORCA‐Flash 4.0 camera (Hamamatsu) and Metamorph software (Molecular Devices) with a 60×/1.4 numerical aperture (NA) oil objective 1 min before and 9 min post‐stimulation with 100 nM agonist, with images acquired at 6‐s intervals. Time‐lapse images were analysed in Fiji ImageJ v1.53c using a macro designed by Imperial College Facility for Light Microscopy. Loss of receptor from the plasma membrane was quantified per frame by calculating full width at half maximum values across the cell membrane and normalised to the average membrane receptor at baseline, and results were expressed as a percentage of internalised receptor over time.

*GLP‐1R recycling*: INS‐1832/3 SNAP‐GLP‐1R cells were seeded in 96‐well imaging plates. Cells were washed twice with PBS and stimulated with 100 nM agonist in complete medium for 1 h to induce receptor internalisation, washed twice with PBS and labelled with 100 nM exendin‐4‐TMR for 1 or 3 h to detect receptor reappearance at the cell surface, followed by a wash in PBS and imaging on a high content Nikon Eclipse Ti2 microscope with a 20×/0.5 NA objective lens, 9 fields of view were acquired per well and analysed in Fiji ImageJ v1.53c. Images were phase segmented, and fluorescence images were background corrected, and fluorescent intensities measured from segmented cell‐containing regions, with measurements normalised to signal from exendin‐4‐TMR‐negative wells.

### 2.11 Ex vivo Ca^2+^ dynamics in mouse islets

Imaging of intact islets 24 h post‐isolation was performed essentially as described.^18^, ^19^ In brief, islets from individual wildtype mice were preincubated for 1 h in Krebs‐Ringer bicarbonate–HEPES (KRBH) buffer (140 mM NaCl, 3.6 mM KCl, 1.5 mM CaCl_2_, 0.5 mM MgSO_4_, 0.5 mM NaH_2_PO_4_, 2 mM NaHCO_3_, 10 mM Hepes, saturated with 95% O_2_/5% CO_2_; pH 7.4) containing 0.1% (w/v) BSA, 6 mM glucose (KRBH G6), and the Ca^2+^‐responsive dye Cal‐520 AM (2 μM, AAT Bioquest). Treatments with KRBH G6 ± 100 nM agonist, 11 mM glucose, or 20 mM KCl were manually added to the islet dishes by pipetting at the indicated time points. To ensure that the islets remained immobile, these were pre‐encased into Matrigel (356 231, Corning) and imaged at 37°C on glass‐bottom dishes (P35G‐1.5‐10‐C, MatTek Life Sciences). Imaging was performed using a Nikon Eclipse Ti spinning disk microscope with an ORCA‐Flash 4.0 camera (Hamamatsu) and Metamorph software (Molecular Devices) with the following settings: λexcitation 488, λemision 510 nm, 20×/0.5 NA air objective. Raw fluorescence intensity traces from whole‐islet ROIs were extracted using Fiji ImageJ v1.53c. Responses were plotted relative to the average fluorescence intensity per islet during the KRBH G6 baseline period, before agonist addition.

### 2.12 Islet insulin secretion assays

Secretion assays were performed in purified mouse and human islets. Human islets from the European Consortium for Islet Transplantation were used for these experiments. Secreted and total insulin were measured essentially as per.^20^ In brief, islets (10/well) were incubated in triplicate for each condition and treatment. Islets (human or mouse) were pre‐incubated for 1 h in KRBH buffer containing 1% (w/v) BSA and 3 mM glucose before incubation with 11 mM glucose ±10 nM agonist in KRBH in a shaking 37°C water bath (80 rpm) for 1 h. Following stimulation, supernatants containing the secreted insulin were collected, centrifuged at 1000 g for 5 min, and transferred to fresh tubes. To determine total insulin contents, islets were lysed using acidic ethanol (75% [v/v] ethanol and 1.5 mM HCl). The lysates were sonicated 3 × 10 s in a water bath and centrifuged at 10 000*g* for 10 min, and the supernatants collected. The samples were stored at −20°C until the insulin concentration was determined using an Insulin Ultra‐Sensitive HTRF Assay kit (62IN2PEG, Cisbio) according to the manufacturer's instructions. GraphPad Prism 9.0 was used for the generation of the standard curve and sample concentration extrapolation. The total insulin content was calculated by adding the secreted insulin to the insulin content of the lysates.

### 2.13 Treatment of *db*/*db* mice

*Db*/*db* mice (C57BL/KsJ‐db/db male/female mice; age 8–11 weeks; body weight 40–60 g; *n* = 8) were randomised based on HbA1c levels. The mice, which were procured from Laboratory Animal Resources (LAR; Sun Pharma Advanced Research Company Ltd.), were housed in individual ventilated cages with free access to food and water and maintained on a 12‐h light/dark cycle. Animals were acclimatised for 3 days. On Day 0, each animal was weighed using a digital weighing balance. Blood was collected by retro‐orbital plexus puncture. Blood glucose level was measured with glucose strips using Blood Glucose Meter (One TouchTM UltraTM; LIFESCAN, Johnson & Johnson) and % HbA1c was measured using A1C Now+® (PTS Diagnostics). Vehicle (placebo), HISHS‐2001 (4.5, 9 and 18 nmol/kg), or tirzepatide (180 nmol/kg) were injected subcutaneously in the neck region of the animals on every third day for 4 weeks (q3d*10). Body weight was monitored. On Day 28 of the study, 24 h following the last dose, blood was collected for measurement of HbA1c, triglycerides (TGs, Colorimetric Assay) and insulin (ELISA).

### 2.14 Measurements of beta and alpha cell mass

*Db/db* mice (C57BL/KsJ‐db/db male/female mice; age 8–11 weeks; body weight 40–60 g; *n* = 8) were randomized based on HbA1c levels. The mice, which were procured from LAR (Sun Pharma Advanced Research Company Ltd.), were housed in individual ventilated cages with free access to food and water and maintained on a 12‐h light/dark cycle. Animals were acclimatized for 3 days. Vehicle (Placebo), tirzepatide (3 and 20 nmol/kg) or HISHS‐2001 (3 and 20 nmol/kg) were injected subcutaneously in the neck region of the animals on every third day for 4 weeks (q3d*10). On Day 28, animals were sacrificed, and the pancreas were collected, fixed with buffered formalin, and paraffin blocks prepared. Six‐micrometre sections of the pancreas were prepared and stained using the Aldehyde‐Fuchsin staining method. After staining, the total section area and area occupied by alpha and beta cells were counted and expressed as relative percent area.

### 2.15 Food consumption

Animals were housed at four mice per cage with pre‐weighed quantities of rodent diet. The amount of food left was measured serially. At the end of the 28‐day treatment period, the amount of diet consumed per cage was determined and represented as food consumption per group.

### 2.16 Pharmacokinetic analyses

Plasma pharmacokinetic study of HISHS‐2001 and tirzepatide was performed in male CD‐1 mice. Ninety animals were procured from LAR. Animals were acclimatized for 1 day. On Day 0, each animal was weighed. Animals were divided into 18 groups (9 groups for HISHS‐2001 and 9 groups for tirzepatide), each containing 5 male animals and representing one time‐point. Animals were injected subcutaneously at 1 mg/kg for the corresponding agonist (dose volume: 10 mL/kg) using 26½ gauge needles. At 1, 2, 4, 8, 12, 24, 48, 72, and 96 h, ~500 μL of blood was collected by retro‐orbital plexus puncture using capillary micro‐centrifuge tubes containing anticoagulant (15 μL 10% K_2_ EDTA). Plasma was separated from blood by centrifugation at ~3300 rpm for 10 min at 4°C. HISHS‐2001 concentrations were estimated using liquid chromatography with tandem mass spectrometry. Lower limit of quantification was 2.14 ng/mL for HISHS‐2001 and 2.04 ng/mL for tirzepatide.

### 2.17 Aversive effects: acute food intake analysis

Lean male C57BL/6 mice were acclimatized to single caging for the study. After overnight fasting, ligands were injected IP at the indicated dose at the beginning of the light phase, and diet was returned. Food consumption was measured by weighing at indicated time points.

### 2.18 Statistics

Data analysis was performed using GraphPad Prism 9.0, involving ANOVA with appropriate post‐hoc correction for multiple testing, and *p* < 0.05 was considered significant; n numbers represent independent biological replicates for in vitro experiments or separate mice for in vivo and ex vivo analyses.

## 3 RESULTS

### 3.1 Receptor pharmacology of HISHS‐2001 resembles that of tirzepatide

**FIGURE 1:**
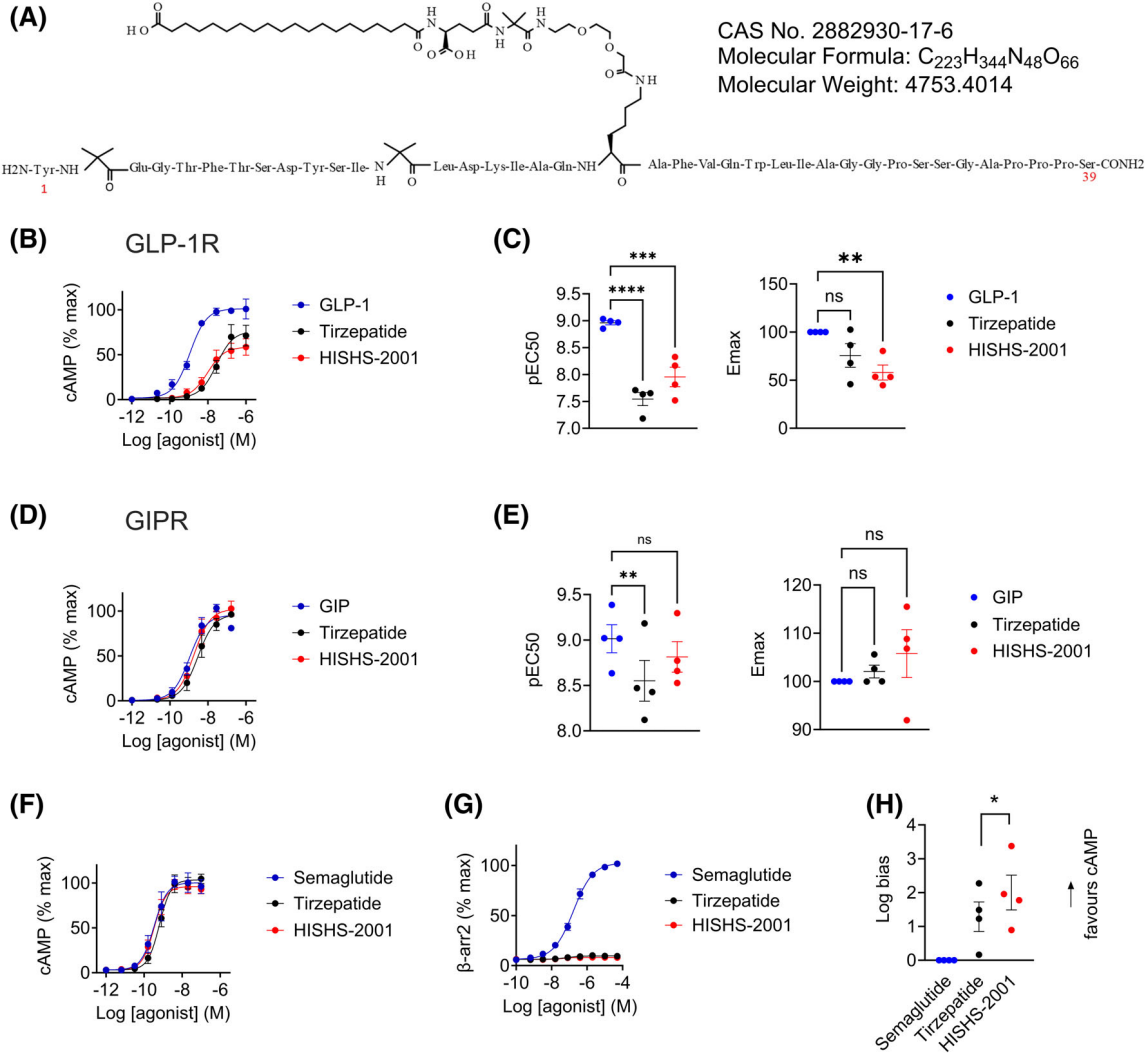
Pharmacological characterization of HISHS‐2001 at GLP‐1R and GIPR. (A) Schematic showing HISHS‐2001 sequence and other formula details. (B) cAMP dose responses for the indicated agonists in HEK293‐SNAP‐GLP‐1R cells, *n* = 4. (C) Potency (pEC50) and maximal response estimates from (B). (D) cAMP dose responses for the indicated agonists in HEK293‐SNAP‐GIPR cells, *n* = 4. (E) Potency (pEC50) and maximal response estimates from (D). (F) cAMP responses in DiscoverX GLP‐1R‐β‐arrestin 2 cells, *n* = 4. (G) β‐arrestin 2 recruitment responses in DiscoverX GLP‐1R‐β‐arrestin 2 cells, *n* = 4. (H) Bias factor calculated using the transduction ratios (*τ*/*K*_A_) method for HISHS‐2021 and tirzepatide compared to semaglutide using data from (F) and (G). Data are shown as mean ± SEM; ns, nonsignificant; *p < 0.05; **p < 0.01; ***p < 0.001; ****p < 0.0001 by one‐way ANOVA with Dunnett's post hoc test.

The structure of HISHS‐2001 is shown in Figure 1A. Similarly to tirzepatide, it consists of a 39 amino acid peptide backbone modified with a fatty acid side chain in Lys 20 and features two α‐amino isobutyric acid residues in Positions 2 and 13. The main difference with tirzepatide lies in the composition of its side chain with a distinct linker sequence that couples the peptide to an albumin‐binding fatty acid moiety.

To explore the biological actions of this molecule, we first examined agonist‐induced cAMP changes in HEK293 cells expressing either SNAP‐tagged human GLP‐1R or GIPR. Both HISHS‐2001 and tirzepatide displayed significantly reduced potency compared to native GLP‐1(7‐36)NH_2_ at the GLP‐1R, although the two synthetic ligands displayed similar potency overall (Figure 1B,C). HISHS‐2001 and tirzepatide both behaved as partial agonists in this system, in keeping with the previously described pharmacology of tirzepatide, which shows reduced maximal ability to activate G_α_s signalling.^21^ Both synthetic agonists showed similar potencies to native GIP(1–42) in GIPR‐expressing HEK293 cells, although, again, tirzepatide was the least potent of all the agonists at the GIPR (Figure 1D,E).

As tirzepatide is reported to be a biased agonist with minimal β‐arrestin recruitment at the GLP‐1R, we next aimed to assess signal bias for both agonists using PathHunter® cells, which allow cAMP and β‐arrestin 2 responses to be determined in parallel. In this cell model, both HISHS‐2001 and tirzepatide showed similar potency to semaglutide for cAMP production at the GLP‐1R, although HISHS‐2001 was moderately more potent (Figure 1F); however, both HISHS‐2001 and tirzepatide showed profoundly reduced ability to drive β‐arrestin 2 recruitment (Figure 1G). Calculation of bias from these data revealed that both dual agonists were strongly biased towards cAMP signalling, with HISHS‐2001 appearing to be the most strongly biased of the two (Figure 1H).

**FIGURE 2:**
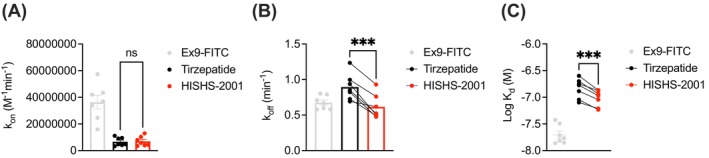
HISHS‐2001 versus tirzepatide GLP‐1R binding kinetics. (A) *K*_on_ calculated from competitive kinetics assay in HEK293‐SNAP‐GLP‐1R cells, *n* = 7. (B) *K*_off_ calculated from competitive kinetics assay in HEK293‐SNAP‐GLP‐1R cells, n = 7. (C) Calculated affinity from data shown in (A) and (B). Data are shown as mean ± EM; ns, nonsignificant; ***p < 0.001 by paired *t*‐test.

Some reports indicate that G protein‐biased GLP‐1R agonists tend to show lower affinity because of faster receptor dissociation kinetics.^20^, ^22^ We therefore used a FRET‐based assay^20^ to determine the binding affinities of each ligand at the GLP‐1R through competitive kinetics with the fluorescent GLP‐1R ligand exendin(9–39)‐FITC (Figure 2). *K*_on_ values for HISHS‐2001 and tirzepatide were similar (Figure 2A), but *K*_off_ (Figure 2B) was significantly lower for HISHS‐2001 than for tirzepatide, such that the calculated K_d_ value (Figure 2C) was also lower. Thus, HISHS‐2001 displays tighter binding at the GLP‐1R than tirzepatide, which could explain its modestly higher potency for cAMP production.

### 3.2 Subtle changes in G_α_s coupling triggered by HISHS‐2001 compared to tirzepatide in pancreatic beta cells

**FIGURE 3:**
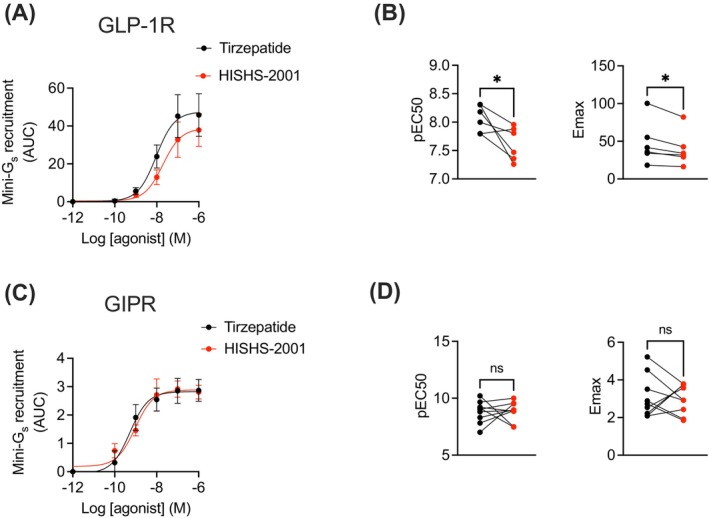
HISHS‐2001 versus tirzepatide G_α_s protein coupling at the GLP‐1R and GIPR in beta cells. (A) GLP‐1R G_α_s recruitment dose response to HISHS‐2001 and tirzepatide at indicated doses by NanoBiT complementation in INS‐1832–3 GLP‐1R^−/−^ cells transfected with GLP‐1R‐SmBiT and G_α_s‐LgBiT. (B) Potency (pEC50) and maximal response from (A); *n* = 6. (C) GIPR G_α_s recruitment dose response to HISHS‐2001 and tirzepatide at indicated doses by NanoBiT complementation in INS‐1832–3 GIPR^−/−^ cells transfected with GIPR‐SmBiT and G_α_s‐LgBiT. (D) Potency (pEC50) and maximal response from (C); *n* = 9. Data are shown as mean ± SEM; ns, nonsignificant; *p < 0.05 by paired *t*‐test.

A NanoBiT complementation assay was next used in INS‐1832/3 rat pancreatic beta cells to explore the coupling between each receptor and G_α_s with both synthetic agonists. Whilst HISHS‐2001 triggered a lower degree of G_α_s recruitment versus tirzepatide at the GLP‐1R (Figure 3A,B), no changes were observed for GIPR coupling to G_α_s with this agonist (Figure 3C,D).

### 3.3 HISHS‐2001 and tirzepatide elicit a similar GLP‐1R trafficking profile and cholesterol‐rich lipid nanodomain partitioning of the receptor

**FIGURE 4:**
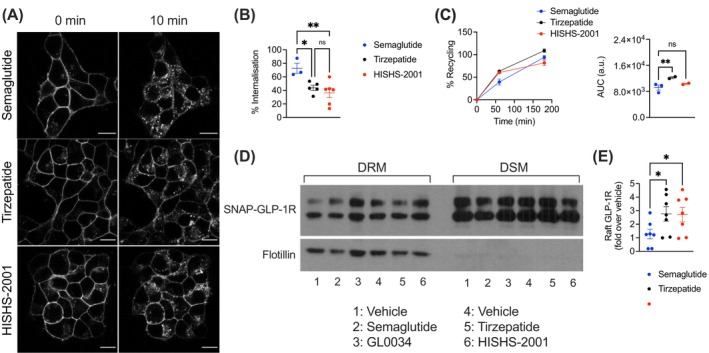
GLP‐1R trafficking profiles of HISHS‐2001 versus tirzepatide or semaglutide in beta cells. (A) Representative images of SNAP‐GLP‐1R subcellular localisation at 0‐ and 10‐min post‐stimulation with 100 nM HISHS‐2001, tirzepatide, or semaglutide in INS‐1832/3 SNAP‐GLP‐1R cells. (B) Percentage of SNAP‐GLP‐1R internalisation with the indicated agonist calculated from (A); *n* = 3–7. (C) Percentage of SNAP‐GLP‐1R recycling to the plasma membrane in INS‐1832/3 SNAP‐GLP‐1R cells in response to 100 nM HISHS‐2001, tirzepatide, or semaglutide, with corresponding AUCs shown; *n* = 2–3. (D) Cholesterol‐rich lipid nanodomain segregation of SNAP‐GLP‐1R in INS‐1832/3 SNAP‐GLP‐1R cells under vehicle conditions or in response to 100 nM HISHS‐2001, tirzepatide, semaglutide, or semaglutide analogue GL0034^20^; DRM, detergent‐resistant membrane fractions; DMS, detergent‐soluble membrane fractions; flotillin indicates cholesterol‐rich lipid nanodomain enrichment. (E) Quantification of SNAP‐GLP‐1R/flotillin from (D); *n* = 7. Data are shown as mean ± SEM; ns, nonsignificant; *p < 0.05; **p < 0.01 by one‐way ANOVA with Dunnett's post hoc test.

GLP‐1R internalization, assessed by confocal imaging of SNAP‐tagged human GLP‐1R stably expressed in INS‐1832/3 cells, revealed slower endocytosis of the receptor following stimulation with both tirzepatide and HISHS‐2001 when compared to the non‐biased GLP‐1R agonist semaglutide (Figure 4A,B), as previously observed for tirzepatide.^23^ A faster GLP‐1R recycling for both receptors versus semaglutide was also observed, although this only reached significance with tirzepatide (Figure 4C).

GLP‐1R signalling efficacy is regulated by recruitment of the receptor to cholesterol‐rich lipid nanodomains.^24^ After exposure to agonists, we employed a biochemical fractionation approach to separate detergent‐soluble and ‐resistant plasma membrane fractions in INS‐1832/3 beta cells stably expressing SNAP‐tagged human GLP‐1R. Western blot analyses of these fractions showed that both tirzepatide and HISHS‐2001 drove similarly elevated incorporation of GLP‐1R into lipid rafts versus semaglutide, with no differences between the two dual agonists (Figure 4D,E).

### 3.4 HISHS‐2001 and tirzepatide elicit similar levels of Ca^2+^ dynamics and potentiation of insulin secretion in primary mouse and human islets

**FIGURE 5:**
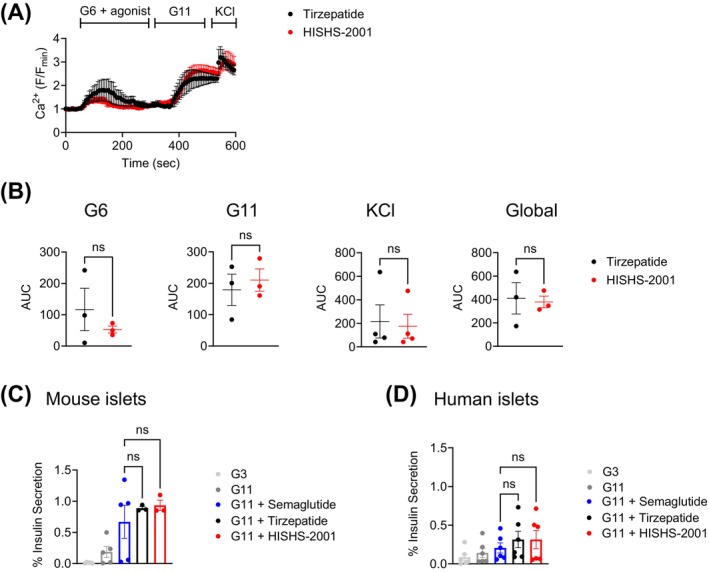
Ex vivo HISHS‐2001 versus tirzepatide downstream functional responses in primary islets. (A) Ex vivo intracellular Ca^2+^ increases in wildtype mouse islets in response to 100 nM HISHS‐2001 or tirzepatide, shown as *F*/Fo (Fo, average baseline signal); G6, 6 mM glucose; G11, 11 mM glucose; KCl, 20 mM. (B) AUCs calculated for the G6, G11, KCl, and global responses; *n* = 3. (C, D) Ex vivo insulin secretion from mouse (C) and human (D) islets at indicated glucose concentrations and in response to 100 nM HISHS‐2001, tirzepatide, or semaglutide; G3, 3 mM glucose; G11, 11 mM glucose; *n* = 3–6. Data are shown as mean ± SEM; ns, non‐significant by paired *t*‐test or one‐way ANOVA with Dunnett's post hoc test.

We next explored the degree of signal transduction elicited by both synthetic dual agonists in primary islets. We first analysed their capacity to elicit intracellular Ca^2+^ rises with the intracellular fluorescent dye Cal520 in islets purified from wildtype mice. Here, both tirzepatide and HISHS‐2001 caused a similar potentiation of glucose‐induced intracellular Ca^2+^ increases, with tirzepatide tending to be more effective at 6 mM glucose, and HISHS‐2001 at 11 mM glucose (Figure 5A,B). We next examined the capacity of both agonists to potentiate glucose‐stimulated insulin secretion in both mouse and human islets, observing a similar potentiating effect by the dual receptor agonists as that promoted by semaglutide at 11 mM glucose in islets from both species (Figure 5C,D).

### 3.5 Effects of HISHS‐2001 on glucose homeostasis in vivo

**FIGURE 6:**
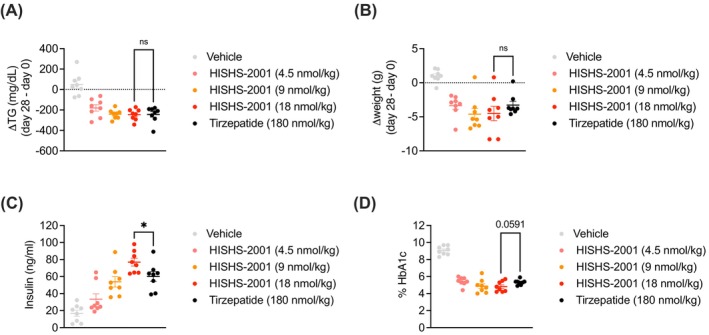
In vivo effects of HISHS‐2001 versus tirzepatide in obese *db/db* mice. (A, B) Change (Day 28 to Day 0) in circulating triglycerides (A) and body weight (B) in obese mixed‐sex *db/db* mice chronically exposed to the indicated agonist; *n* = 8. (C, D) Plasma insulin (C) and % HbA1c (D) levels measured at Day 28 in mice from (A, B). Data are shown as mean ± SEM; ns, nonsignificant; *p < 0.05, by one‐way ANOVA with Sidak's post hoc test.

Finally, a chronic in vivo study was performed where obese mixed sex *db/db* mice were exposed to HISHS‐2001 or tirzepatide for 28 days. The stability of each drug in the circulation was similar (Table S2). We also observed similar reductions in circulating TGs and body weight with both agonists (Figure 6A,B), but with HISHS‐2001 used at a 10 times lower dose compared to tirzepatide. The effects of each drug on food intake were similar (~50% lowering; Table S3), and both agonists also caused similar reductions in HbA1c with HISHS‐2001 used at a 10‐fold reduced dose, albeit with a near significant improvement in this parameter in favour of HISHS‐2001. Furthermore, increases in circulating insulin were significantly greater for HISHS‐2001 despite the reduced dosage versus tirzepatide (Figure 6C,D).

These differences were further explored using lower doses of tirzepatide (Figure S1). At 3 nmol/kg, no differences were observed in the efficacy of HISHS‐2001 and tirzepatide on circulating TG, weight, or circulating insulin, though the former was significantly more effective in lowering HbA1c at this dose. At the higher (20 nmol/kg) dose of each drug, HISHS‐2001 was similarly effective to tirzepatide on controlling TG and weight but was significantly more effective in controlling HbA1c and releasing insulin (Figure S1). We note that similar differences between the actions of the two ligands on insulin secretion were not, however, observed in ex vivo studies with human islets (Figure 5D).

### 3.6 Effects of drug treatment on islet cell mass

HISHS and tirzepatide had similar overall effects on increasing alpha and beta cell mass compared to Vehicle treatment in pancreases from *db/db* mice under the same conditions as those explored above (Figure S2), with alpha:beta cell mass ratios similar under each treatment.

### 3.7 Assessment of hyperacute anorectic effects in vivo

**FIGURE 7:**
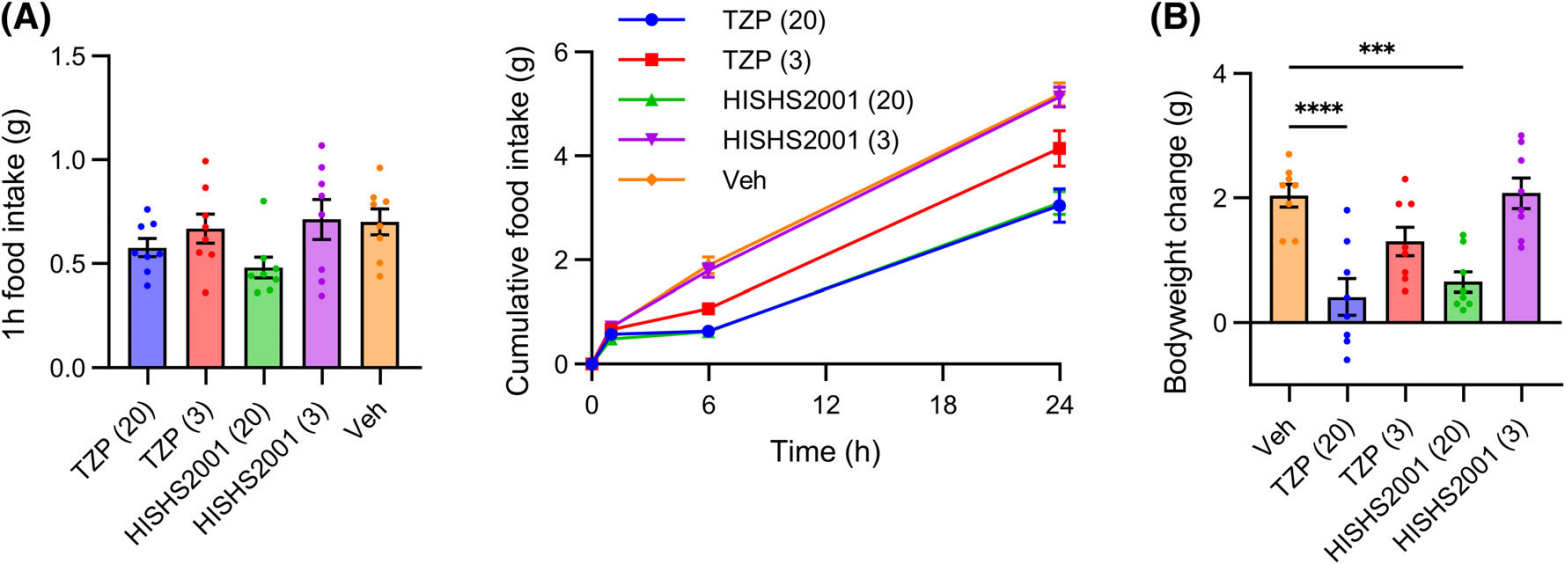
Anorectic effects of HISHS‐2001 in lean mice. (A, B) Cumulative food intake (A) and change in body weight (B) in overnight‐fasted mice (*n* = 8 per group) injected with the indicated dose in nmol/kg of HISHS‐2001 or tirzepatide at the beginning of the light phase. Data are shown as mean ± SEM. Food intake is compared by two‐way repeated measures ANOVA with Dunnett's test versus vehicle, with * indicating statistical significance (*p* < 0.05) for the agonist of the same colour Weight gain is compared by one‐way ANOVA with Dunnett's test versus vehicle. ***p < 0.001, ****p < 0.0001.

GLP‐1R agonist treatment can cause nausea at high doses, but there is some evidence that concurrent GIPR agonism can reduce nausea. Due to differences in the emesis systems between humans and mice, as a surrogate for nausea we measured the effect of TZP and HISHS2001 to hyper‐acutely suppress food intake in overnight‐fasted mice. At both 3 and 20 nmol/kg, neither ligand led to a significant reduction in food consumption in the first hour, although at the higher dose both ligands suppressed food intake and reduced the restoration of post‐fasting body weight at 24 h (Figure 7). The relatively slow onset of appetite suppression with these ligands, at doses that produce clear metabolic effects during chronic treatment, raises the possibility of a somewhat reduced nausea potential. However, species differences mean firm conclusions cannot be drawn.

## 4 DISCUSSION

We have recently described a novel GLP‐1R agonist, utreglutide (GL0034), with improved metabolic effects versus semaglutide.^20^ Here, we sought to develop a similar, long‐acting dual GLP‐1R/GIPR receptor agonist and compare it to tirzepatide. We show that HISHS‐2001 (GL0059) displays higher GLP‐1R affinity and bias towards cAMP production over β‐arrestin 2 recruitment, as well as comparable or improved metabolic effects in the *db/db* mouse model of diabetes versus tirzepatide.

The main aim of the present study was to synthesize a novel GLP1R‐GIPR dual agonist and perform its functional characterization in vitro and in vivo. We show that HISHS‐2001 has several favourable characteristics, including a higher affinity at the GLP‐1R and a more pronounced cAMP over β‐arrestin 2 biased signalling profile when compared to tirzepatide. We also observed small apparent differences in the G protein coupling abilities of HISHS‐2001 and tirzepatide at the GLP‐1R. On the other hand, signalling towards intracellular Ca^2+^ changes, chiefly involving alterations in plasma membrane potential and Ca^2+^ influx though voltage‐gated calcium channels,^25^ with little or no mobilization via G_α_q‐coupled receptors,^26^ was not different between the two agonists. The changes in signalling bias described above were associated with only subtle differences in GLP‐1R trafficking and no difference in cholesterol‐rich nanodomain recruitment of the receptor in pancreatic beta cells, as well as no significant differences in the ex vivo capacity of these agonists to potentiate insulin secretion in mouse or human islets.

We note that the responses of human islets to stimulation both with elevated glucose and incretin receptor agonists were somewhat weaker than those observed in mouse islets, in large part reflecting the complexity and inevitable delays involved in the isolation of human versus mouse islets, and probably related to cold ischaemia time and deterioration in function which accompanies islet transportation between sites of isolation and experimentation. Nevertheless, the patterns of response to the tested compounds were similar in islets from each species, supporting our conclusions regarding the comparable efficacy of HISHS‐2001 versus tirzepatide with respect to their capacity to stimulate insulin secretion in humans.

Importantly, however, we note that differences may exist between the contribution of GLP‐1R and GIPR signalling for the effects of tirzepatide in mouse versus human, and that the apparent advantages of GLP‐1R/GIPR co‐agonists in mice may simply reflect altered signalling properties (e.g., bias) at the GLP‐1R.^27^ Nevertheless, others have shown the importance of GIPR signalling for the effects of tirzepatide in mice, which exceed those of semaglutide,^28^ and as such our use of a mouse model here is valid to demonstrate the non‐inferiority of HISHS‐2001 versus tirzepatide in vivo. Importantly, we provide validation for our findings using the abovementioned insulin secretion studies in human islets, where responses to HISHS‐2001 closely correlated with those observed in mouse islets. Future human‐centric in vivo studies, involving the use of mice humanised for the GIPR, and ultimately studies in humans beyond the scope of the present report, will be required to fully assess the effects of GLP‐1R/GIPR co‐agonists such as HISHS‐2001.

We also note that differences in bias between HISHS‐2001 and other receptor agonists might impact efficacy in certain physiological contexts and may have implications for other signalling pathways and the mechanisms of action of the drugs.

Importantly, explored in vivo in a mouse model of obesity‐related diabetes (*db/db*), HISHS‐2001 was equally effective as tirzepatide in reducing TG levels and body weight, even though the latter drug was used at a considerably higher dose. Moreover, HISHS‐2001 was more effective than tirzepatide at increasing circulating insulin levels and tended to cause a more marked lowering in HbA1c, indicating that our in vitro findings can be extended to the in vivo setting. Although this could conceivably reflect differences in the pharmacokinetic profile of the two drugs, our preliminary measurements (Table S2) failed to reveal a marked difference in this parameter. In any case, this explanation would seem unlikely given the higher dose of tirzepatide used in our study.

The effects of both drugs on body weight are likely, at least in a large part, to reflect a reduction in appetite and feeding (Table S3). These changes are presumably the result of actions at feeding centres in the brain, which may include agouti‐related protein neurons in the arcuate nucleus^29^ as well as other neuronal populations.^30^ Central effects of GLP‐1R (and GIPR) agonism may also be involved more directly (i.e., independently of weight loss, but involving changed neural inputs to the endocrine pancreas) in the actions of HISHS‐2001 to increase insulin secretion.^31^, ^32^ We note that the contribution of GIPR agonism to the effects of HISHS‐2001 and other co‐receptor agonists in vivo remains to be resolved given that antagonism at this receptor also promotes weight loss.^33^ However, differences in signalling bias between HISHS‐2001 and tirzepatide at the GLP‐1R, as demonstrated here in studies in model cell systems, do not appear to be important in central neurons in controlling body weight. Future studies, involving investigations of food intake and energy expenditure outside the scope of the present report, will be needed to explore these questions in more detail.

## 5 CONCLUSION

We suggest that HISHS‐2001 provides an attractive dual receptor agonist which may be useful clinically as an antihyperglycaemic agent in human obesity and T2D.

## CONFLICT OF INTEREST STATEMENT

Vinod Burade, Thennati Rajamannar, Muthukumaran Natarajan, and Pradeep Shahi are employees of Sun Pharmaceuticals, from whom Guy A. Rutter and Alejandra Tomas have received grant funding.

## PEER REVIEW

The peer review history for this article is available at https://www.webofscience.com/api/gateway/wos/peer-review/10.1111/dom.16652.

## Supporting information


**Data S1.** Supporting Information.

## Data Availability

All data are available in the main article and/or the Supporting Information.
