# Supplementary material for: Binding kinetics, bias, receptor internalization and effects on insulin secretion in vitro and in vivo of a novel GLP‐1R/GIPR dual agonist, HISHS‐2001
Source: Diabetes Obes Metab. 2025 Aug 20;27(10):5938–49. doi: 10.1111/dom.16652 (PMC12409219; doi:10.1111/dom.16652)
Supplement: Supplementary file 1 — Data S1. Supporting Information. [file DOM-27-5938-s001.docx]

**Supplementary Information**

Manchanda *et al.*, Binding Kinetics, Bias, Receptor Internalization and Effects on Insulin Secretion *in vitro* and *in vivo* of a Novel GLP-1R/GIPR Dual Agonist, HISHS-2001

**Table S1: Human islet preparations used in the study.** COD, cause of death; CVD, cardiovascular disease.

| **Age (Years)** | **Sex** | **BMI (Kg/m^2^)** | **HbA1c (%)** | **COD** | **Origin** |
| --- | --- | --- | --- | --- | --- |
| 63 | M | 29.4 | - | CVD | Pisa |
| 76 | F | 23.9 | - | CVD | Pisa |
| 32 | M | 24.8 | 4.5 | - | Alberta |
| 47 | M | 40.3 | 5.5 | - | Alberta |
| 64 | F | 22.07 | - | CVD | Pisa |
| 53 | F | 27.2 | - | - | Milan |

**Table S2: Pharmacokinetic analysis of HISHS-2001 *versus* tirzepatide.** See main manuscript text for further details.

| **Treatment 30 nM/kg**  **(N=5)** | **AUC_0-t_**  **(hr*ng/mL)** | **AUC_0-∞_**  **(hr*ng/mL)** | **C_max_**  **(ng/mL)** | **T_max_**  **(hr)** | **T_1/2_**  **(hr)** | **K_el_**  **(1/hr)** |
| --- | --- | --- | --- | --- | --- | --- |
| **HISHS-2001** | 17307.8 | 17345.2 | 779.90 | 8.0 | 10.09 | 0.069 |
| **Tirzepatide** | 14080.6 | 14090.3 | 685.85 | 8.0 | 8.78 | 0.079 |

**Table S3: Effects of HISHS-2001 *versus* tirzepatide on cumulative food intake.** See main manuscript text for further details.

| **Treatment** | **Cage No.** | **Food intake (g), Day 28 (N=4)** | **Mean (g)** | **SD** |
| --- | --- | --- | --- | --- |
|  |  |  | **N=8** | |
| **Diabetic Control** | 1 | 212.4 | 202.5 | 14.00 |
|  | 2 | 192.6 |  |  |
| **HISHS-2001**  **4.5 nM/kg (q3d*10)** | 3 | 116.85 | 112.4 | 6.28 |
|  | 4 | 107.975 |  |  |
| **HISHS-2001**  **9 nM/kg (q3d*10)** | 5 | 108.2 | 111.8 | 5.11 |
|  | 6 | 115.425 |  |  |
| **HISHS-2001**  **18 nM/kg (q3d*10)** | 7 | 78.55 | 89.5 | 15.49 |
|  | 8 | 100.45 |  |  |
| **Tirzepatide**  **180 nM/kg (q3d*10)** | 9 | 87.65 | 90.1 | 3.43 |
|  | 10 | 92.5 |  |  |


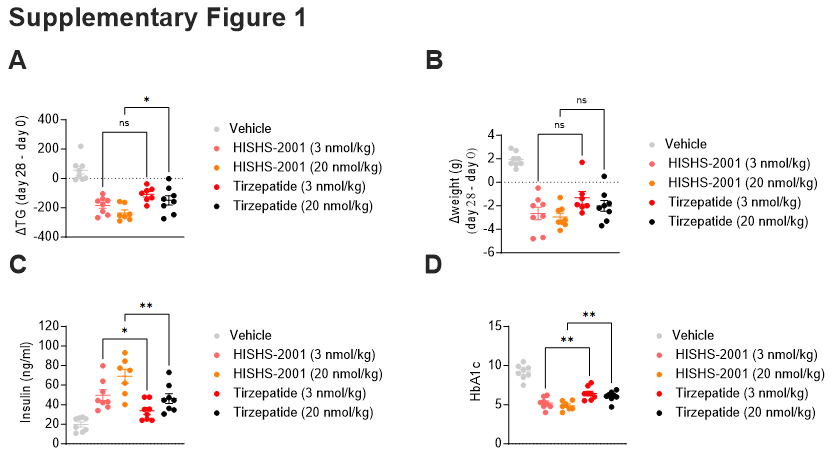


***In vivo* effects of high and low dose HISHS-2001 *versus* tirzepatide in obese *db/db* mice.** (**A**, **B**) Change (day 28 – day 0) in circulating triglycerides (**A**) and body weight (**B**) in obese mixed sex *db/db* mice chronically exposed to the indicated agonist; n=7-8. (**C**, **D**) Plasma insulin (C) and HbA1c (D) levels measured at day 28 in mice from (A, B). Data is shown as mean +/- SEM; ns, non-significant; *p<0.05, by one-way ANOVA with Sidak’s post-hoc test.
